# Contributions of a blended learning based on peer evaluation for teaching drug-drug interactions to undergraduate pharmacy students

**DOI:** 10.1186/s12909-019-1867-5

**Published:** 2019-11-19

**Authors:** Roland Lawson, Hélène Géniaux, Serge Bailly, Christelle Pouget, Catherine Fagnère, Marie-Laure Laroche, Jacques Monteil, Jean-Jacques Moreau, Nicolas Picard

**Affiliations:** 1 Département de pharmacologie, Univ. Limoges, Faculté de Pharmacie, Inserm U1248, F-87000 Limoges, France; 2 Faculté de Pharmacie, Université de Limoges, 2 rue du Dr Marcland, 87025 Limoges, France; 3 CHU Limoges, Centre régional de pharmacovigilance, de pharmaco-épidémiologie et d’information sur les médicaments, F-87000 Limoges, France; 4 Service commun d’ingénierie pédagogique (UL Community), University of Limoges, F-87000 Limoges, France; 5 Département de chimie organique et thérapeutique, , Faculté de Pharmacie, University of Limoges, F-87000 Limoges, France; 6 Département de pharmacologie, Faculté de Médecine, University of Limoges, F-87000 Limoges, France; 7 Département Universitaire d’Enseignement Numérique en Santé (DUENES), Faculté de Médecine, University of Limoges, F-87000 Limoges, France

**Keywords:** Blended learning, Peer-evaluation, Moodle, Adverse drug reaction, Drug-drug interaction

## Abstract

**Background:**

Numerous studies have pointed out the need for better training of healthcare professionals in drug-drug interactions management in order to minimize adverse drugs reactions impacts on patients. The aim of this study was to evaluate the benefits of a blended learning strategy based on peer evaluation (PE) for teaching drug-drug interactions to undergraduate pharmacy students.

**Methods:**

Third-year pharmacy students (*n* = 72) from the University of Limoges were involved in a hybrid teaching using the Moodle platform (2.9 version). After the theoretical lectures, an online activity was proposed to students. Each student submitted a report addressing a clinical case for peer evaluation. Students evaluated the pedagogical approach using an online survey. Quantitative benefits were assessed from students randomly assigned into two groups: PE in pharmacodynamics items (PE-PD) or PE in pharmacokinetics items (PE-PK). During this activity, three marks were given: one from peers for their evaluation work and two from teachers for oral group presentation of the clinical cases and for the final written examination. Statistics were performed using two-tailed unpaired t-test and significance was set for *p* < 0.05.

**Results:**

Only a few students (*n* = 14, 20.6%) were aware of the peer evaluation principle and even less, only one student (n = 1, 1.5%), had already encountered it. Students considered that they benefited from this evaluation (*n* = 65, 95.6%); from their work being reviewed (*n* = 62, 91.2%) and that they participated in improving their classmates understanding (*n* = 59, 86.8%). Peers’ allocated marks were similar in the two PE groups (PE-PD = 17.4 ± 1.4; PE-PK = 17.3 ± 1.4). Teachers’ marks for oral presentation were significantly lower for pharmacodynamics than for pharmacokinetics items (PE-PD = 15.2 ± 1.2; PE-PK = 16.1 ± 2.1; *p* < 0.05). The final examination marks were equivalent in both groups (PE-PD = 11.0 ± 2.1; PE-PK = 11.2 ± 1.9).

**Conclusions:**

Besides the fact that a major short-term quantitative improvement was not detected, our teaching approach was qualified as being a positive and stimulating learning tool by students.

## Background

Adverse drugs reactions (ADRs) can range from minor discomfort to severe events leading to hospitalization, permanent disability or even death [1]. Several of these reactions are related to drug-drug interactions (DDI). Numerous studies have pointed out the need for better training of healthcare professionals in pharmacological background of ADRs and DDI management [2, 3]. Healthcare professionals should acquire strong background knowledge to be capable of dealing with complex clinical cases, and develop skills to be able to rationally prescribe, administer and monitor drug therapy. A recent review of the literature highlighted the urgent need to modernize clinical aspects of pharmacovigilance education (ADRs, DDI) in healthcare curricula by offering real-life training to students [4]. In this context, we decided to investigate the potential benefits of an interactive pedagogical approach similar to the professional situations, based on peer evaluation (PE) for improving professional skills for pharmacy students.

Blended learning is referred to as a hybrid teaching methodology and is the combination of traditional in-person classroom activities and structured independent online studying periods guided by a facilitator [5]. Blended learning has been found to improve clinical skills of healthcare students [6]. In this study, this approach was associated with peer evaluation, which is essential to science and medicine: PE has been used as an editorial tool since the eighteenth century and was generalised in the mid twentieth century for the vast majority of scientific journals. Although PE is a selection process in science which is used prior to publishing research results, it can also be used for student assessments [7, 8]. It is based on the theory of cognitive congruence and social constructivism [9]. According to K. J. Topping, “peer assessment is an arrangement for learners to consider and specify the level, value or quality of a product, or performance of other equal-status learners. Products to be assessed can include writing, oral presentation, portfolios, test performance, or other skilled behaviours” [10]. This approach reduces the considerable gap in knowledge between a student and his teacher in favour of a relatively smaller gap between students who help each other to learn. Peer assessment is widely used on a large scale for Massive Open Online Course (MOOC), but can also be adapted for small groups of students. This approach, which promotes interaction among peers, is also known to improve self-esteem and commitment to work, and to overcome personal fears and lack of confidence with positive outcomes during healthcare training [11].

Our study aims to evaluate the benefits of a blended learning based on a PE approach in the field of pharmacological background of ADRs and DDI among pharmacy students. We analysed subjective feedback from students using an online questionnaire and then evaluated the quantitative benefits of this approach based on the different marks allocated by peers and teachers.

## Methods

### Study population and sample size

The study was conducted at the University of Limoges over two consecutive academic years in order to reach an appropriate sample size with undergraduate third-year pharmacy students (*n* = 72).

### Overview of the University of Limoges and its pharmacy program

The University of Limoges located in the region Nouvelle-Aquitaine, is a French national multidisciplinary university with more than 16,000 students awarding licenses, masters and doctorates in all traditional knowledge sectors and innovative fields. In order to put an accent on implementing digital technologies in pedagogy, a digital health education department has been created to meet the various challenges of initial training learners in health science. The School of pharmacy delivers a degree of Doctor in Pharmacy after a minimum of 6 years training. Undergraduate pharmacy education in France is similarly organised in all the 24 French schools of pharmacy. The first year dedicated to basic knowledge in health science is common with medicine, pharmacy, odontology and midwifery. The curriculum is then divided in 3 cycles. The first cycle includes the second and third years and is dedicated to basic scientific knowledge in biology, physics, mathematics, chemistry physiology and public health. The second cycle includes the fourth and fifth years and is based on coordinated teaching, involving several disciplines and self-learning. The third cycle includes the sixth year for students destined for careers as pharmacists in community pharmacy, pharmaceutical industries or 3 additional years for hospital-based careers [12].

### Study design

This activity was divided into 3 parts. The first part followed the information meeting and was related to theoretical pharmacological background of ADRs knowledge taught in-person or online by a teacher. This was followed by a second step of distance working using the Moodle® 2.9 platform. The last part was a classroom-based activity. Moodle® was used to organize the progression. Using this platform, students had continuous access to lectures, pedagogical external links, a discussion forum, as well as timetables.

After the theoretical lectures, an online analysis of clinical cases (Additional file 1) was proposed. Excel random function was used to blindly generate diverse groups of 4 students. All the students in the same group were assigned the same clinical case. Students had to submit online, an anonymous individual structured report addressing the clinical case, following the teachers’ instructions, within a fixed period of 6 days. The students’ works were then blindly assigned for assessment by three peers according to a scoring grid (Additional file 2). The peers were assigned a different clinical case to the ones they had to assess. Peers’ work was performed anonymously. The deadline for submitting peer assessment was 6 days. After this step, the students were told which group they were in and each group was reconstituted for a classroom discussion in order to improve their work collectively under the supervision of teachers who could give them advice (Table 1).

**Table 1:** Study population and design

Study population		Undergraduate, third-year pharmacy students (*n* = 72)
Study design	Information meeting	Explanation of the objectives and different activities proposed in this module
	Theoretical lectures	Online or in-person
	Distance working	• Moodle® platform (2.9 version) for access to lectures, pedagogical external links, a discussion forum and timetables. • Blind inclusion of learners in groups of 4 students (Excel random function). • Assignment to each group of different clinical cases for analysis during 6 days. • Individual submission of a structured report addressing the case following a precise plan and instructions given by teachers. • Blind peer evaluation of three different submissions from the one previously assigned to each reviewer with a deadline of 6 days
	Classroom-based activity	Students are divided into their respective groups for classroom work and discussion in order to improve their work collectively under the supervision and guidance of teachers. Oral group presentation in front of all the students
	Evaluation of the benefits of the pedagogical approach	Analysis of student perceptions via an online survey using Google Forms® platform.
		Final written examination for quantitative evaluation on knowledge acquisition

### Data collection

**Fig. 1:**
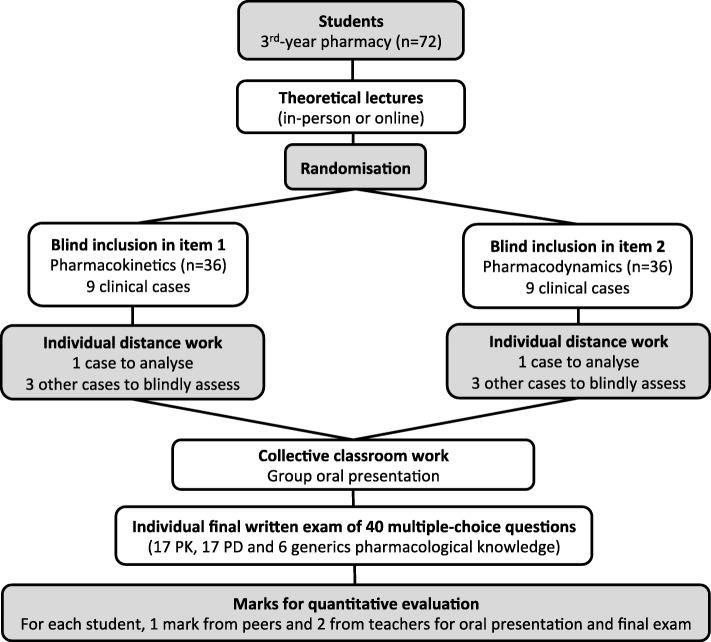
Flowchart of the quantitative evaluation of the pedagogical experience

Students’ perceptions of this learning approach were assessed using an online questionnaire accessible on an independent platform (Google Forms®) in order to guarantee anonymity (Additional file 3). To allow quantitative performance evaluation, students randomised in groups, were assigned pharmacology ADRs clinical cases related either to pharmacokinetics (PK) or pharmacodynamics (PD) situations. Peers only reviewed works related to the subject they were assigned (PK or PD). The classroom discussion was followed by an oral group presentation of each clinical case in front of all the students. Students were given three different marks during this activity: one from peers and two from teachers (one related to the oral presentation and the other one for the final individual PD-PK exam). Teachers used the same rubrics of the scoring grid of the PE to evaluate the oral presentations. The final written examination was based on 40 multiple-choice questions (MCQs) (17 related to PK, 17 to PD and 6 to generic pharmacology knowledge) (Fig. 1). All the students were marked out of 20.

### Statistical analysis

Descriptive data are shown as proportions for categorical variables and mean ± standard deviation for scaled responses. Quantitative results are expressed as mean ± standard deviation. Statistical comparisons between groups were made using a two-tailed unpaired t-test. Statistical analyses were done using GraphPad Prism (version 6.01) with a significance threshold set to *p* < 0.05.

### Ethics and consent

According to the French legislation, submission to an ethics committee is not mandatory for our study. All the students were informed about the objectives of this pedagogical investigation before starting the activity and signed a written informed consent to release their grades for education research. The participation in the online questionnaire was voluntary.

## Results

### Effects of the blended learning strategy on knowledge assessment

#### Peers’ marks and teachers’ oral presentation marks

**Fig. 2:**
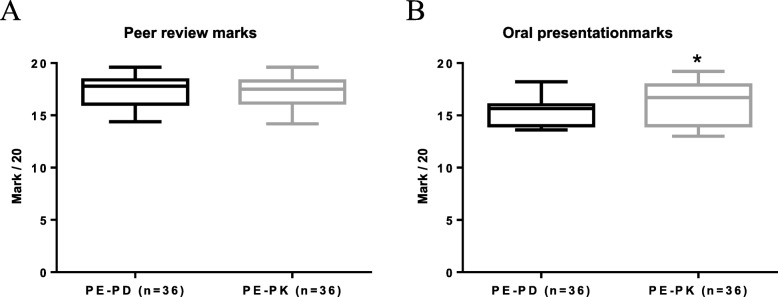
Peer evaluations and oral presentations marks. **a** Peers marks for individual production were similar (*p* > 0.05; unpaired t-test) for both PE-PD (median 17.8; min 14.4; max 19.6) and PE-PK (median 17.5; min 14.2; max 19.6). **b** Teachers’ oral presentation marks were lower than peers’ previous marks and significantly lower (*p < 0.05; unpaired t-test) for PE-PD (median 15.7; min 13.6; max 18.2) than PE-PK (median 16.7; min 13; max 19.2). PE-PD = group of peer evaluation in pharmacodynamics item; PE-PK = group of peer evaluation in pharmacokinetics item; n = number of students

Peers marks for individual production were similar for both PE-PD (median = 17.8; min = 14.4; max = 19.6) and PE-PK groups (median = 17.5; min = 14.2; max = 19.6). However, teachers’ marks after oral group presentations were significantly lower (*p* < 0.05) for the PE-PD group (media*n* = 15.7; mi*n* = 13.6; max = 18.2) than the PE-PK group (median = 16.7; min = 13; max = 19.2) (Fig. 2).

#### Final examination

The score of the final examination was equal for PD and PK. The marks for generic questions were also similar: PE-PD (media*n* = 11.7; mi*n* = 9.5; max = 17.0) and PE-PK (median = 12.8; min = 8.0; max = 16.0) inferring that the two randomized groups had an equivalent background in pharmacology.

**Fig. 3:**
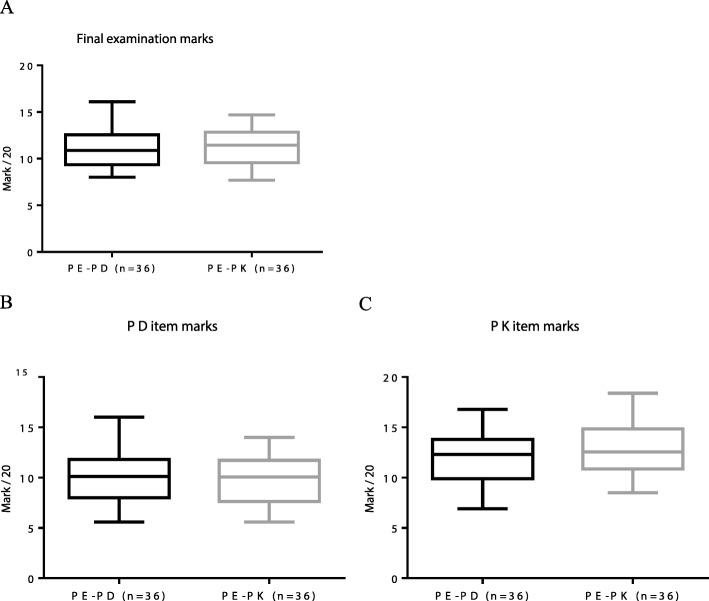
Final examination marks. **a** Global final exam marks showing no difference between PE-PD (median 10.9; min 8.0; max 16.1) and PE-PK (median 11.5; min 7.7; max 14.7) groups. **b** Pharmacodynamics related multiple choice questions marks at final exam were not significantly different for PE-PD (median = 10.1; min = 5.6; max = 16.0) and PE-PK (median = 10.0; min = 5.6; max = 14.0) p > 0.05 unpaired t-test. **c** Pharmacokinetics related multiple choice questions marks at final exam were not significantly different for PE-PD (median = 12.3; min = 6.9; max = 16.8) and PE-PK (median = 12.6; min = 8.5; max = 18.4) p > 0.05 unpaired t-test. PE-PD = group of peer evaluation in pharmacodynamics item; PE-PK = group of peer evaluation in pharmacokinetics item; n = number of students

The final marks were lower and more discriminatory than the marks given by the peers and for the oral presentations. There was no difference between PE-PD (median = 10.9; min = 8.0; max = 16.1) and PE-PK (median = 11.5; min = 7.7; max = 14.7) marks. PD related multiple choice questions marks were not significantly different for PE-PD (median = 10.1; min = 5.6; max = 16.0) and PE-PK (median = 10.0; min = 5.6; max = 14.0) groups. Moreover, PK related multiple choice questions scores were not significantly different for PE-PD (median = 12.3; min = 6.9; max = 16.8) and PE-PK (median = 12.6; min = 8.5; max = 18.4) groups while the marks in PD were lower than those obtained in PK, as previously observed for the oral presentation marks (Fig. 3).

### Peer evaluation based teaching was a new pedagogical experience

PE-based teaching was a totally new approach for the majority of the students. According to the online survey, which was filled in by 94.4% (*n* = 68), only 20.6% (*n* = 14) knew the general principle of PE before starting the training (Table 2). Among the 14 students who knew about the PE principle, 7 were aware of its use in pedagogy and 2 had already been involved in this approach but only 1 person in the field of pedagogy.

**Table 2:** Learners knowledge of peer evaluation principles before the training and opinions about resources and course activities

Items of the questionnaire		Students (94.4% feedbacks, n = 68 under 72)
Principles of peer evaluation (PE) Students who …	knew the general principles	20.6% (n = 14)
	knew principles applied to pedagogy	13.2% (n = 9)
	had pedagogical experience	1.5% (n = 1)
Learning resources and course activities Students who …	struggled with IT tools	16.2% (n = 11)
	found instructors explanations prior to the course not clear enough	0% (*n* = 0)
	found the period of time to produce the work too short	1.5% (n = 1)
	found reviewing work to be constraining	22.1% (n = 15)
	found the period of time to review peers works too short	2.9% (n = 2)
	found the number of works to review too important	10.3% (n = 7)
	found the scoring grid not well adapted	19.1% (n = 13)

The basic computer skills of the students were adequate but some of them struggled with the IT tools used in this teaching experience (16.2%, *n* = 11). The main problems concerned the upload and download functions of the Moodle® platform. The teachers explanations about the PE principle was judged as being very clear for all the students.

### Learner opinions or criticisms about the course activities

In a scale ranging from 0 to 10, the overall feeling about this activity was ranked 8.0 ± 1.0. Although the students were very enthusiastic about this teaching experience some of them judged their work as a reviewer to be constraining (22.1%, *n* = 15) and stated that their own knowledge was not sufficient enough to be able to judge their peers’ works (33.8%, *n* = 23). The majority stated they were able to review a mean of 4 works with in the due date against 3 assigned in this activity and that it was rewarding to be called upon as a reviewer (55.8%, *n* = 40). However, the number of 3 works assigned to peers for evaluation was judged to be too important for 10.3% (*n* = 7). Overall, the classroom collaborative group-working step was found to be either useful or indispensable for 95.6% (*n* = 65).

### Student perceptions

This teaching approach contributed in various ways to learning according to the students. The reviewers thought that their evaluation work could not only contribute to their peers learning experience (86.8%, *n* = 59), but also to their own learning (91.2%, n = 65). In addition, peers felt that being evaluated helped to improve their learning experience (95.6%, n = 65) (Table 3).

**Table 3:** Learners perceptions about pedagogical benefits of the training

Items of the questionnaire		Students (94.4% feedbacks, n = 68 under 72)
Reviewers thought that …	their work improved their peers learning experience	86.8% (n = 59)
	their work improved their own learning experience	91.2% (n = 62)
Peers thought that …	reviewers improved their own learning experience	95.6% (n = 65)

Benefits from this teaching approach were mostly identified as follows: helpful in memorizing knowledge (39.7%, *n* = 27); a better understanding of knowledge (55.9%, *n* = 38); questioning the students own work (64.7%, *n* = 44); acquiring additional knowledge (70.6%, *n* = 48) and critical thinking skills (72.1%, *n* = 49).

## Discussion

Pharmacology ADRs and DDI managements are important missions for pharmacists and healthcare professionals in general requiring both general knowledge and analytical skills. According to our survey, students who participated in this pedagogical experience found the combination of blended learning and peer evaluation innovative and were mainly positive about the potential of this approach in learning outcomes. The pedagogical format was also found to enable collaborative and self-directing learning that are crucial in professional life. In our study, teachers’ marks for oral presentation and final examination, which were more discriminatory than the students’, revealed potentially more difficulties in learning pharmacodynamics than pharmacokinetics. This observation also evident the variation of marking at different experience levels. According to our quantitative study, selective experience of this approach in PD or PK subjects did not show a significant short-term impact in terms of marks. However, previous studies had demonstrated that blended learning approaches were effective in optimizing student learning and in improving performances in health sciences courses [13]. For example, in a basic PK teaching program, the blended learning approach was found to increase students enthusiasm and commitment [14]. According to the current literature, the peer evaluation in health professions education contributes positively to enhancing skills to work in multidisciplinary teams, increasing students’ confidence and quality of work. However, some studies have pointed out the lack of expertise in making assessments [15, 16]. In our experiments, in which only a few students have the concept of peer evaluation, the consistency and reliability of the individual response is somehow a question of personal interpretation of the scoring grid. At this level, peer allocated marks are not discriminatory enough to replace the final exam, which aims to verify knowledge acquisition.

This study was designed to protect against potential biases that could compromise its outcomes. The online survey was accessible anonymously and a randomisation process was used to avoid inclusion bias during the quantitative study evaluating the impact on student performance. Six generic questions introduced into the final MCQ exam showed that the two randomized groups (PE-PD and PE-PK) had an equivalent background in pharmacology.

Despite the methodology used for this study, there are a few limitations to take into account. The use of a scoring grid was supposed to direct student performance by improving self-efficacy. However, largest gains are often found after longer or larger interventions [17]. The fact that the pedagogical format was new, could possibly underestimate the potential impact on the study outcomes. Due to the anonymous survey, we were not also able to analyse relation to performance for students that were more positive on the impact of the blended strategy to see whether they perform better or overestimate themselves.

## Conclusions

Our study provides the potential benefits of a blended learning strategy in the field of DDI teaching at undergraduate pharmacy studies level mainly based on the students’ perceptions. The short-term quantitative effects were probably moderated because this pedagogical approach was new to our study population.

## Supplementary information

**Additional file 1.** Examples of clinical cases. Two typical clinical cases related to pharmacokinetics and pharmacodynamics topics addressed by students during this training are presented.

**Additional file 2. **Scoring grid (implemented in Moodle**®** platform). From this grid, peer evaluation mark obtained from each student under a total of 32 points and then reported under 20. PE-mark = [Σ (item mark* weight) * 20]/32

**Additional file 3.** Online survey. Items of the survey completed by students after the training to evaluate relevance and usefulness of the blended learning and peer review approach in teaching drug-drug interactions.

## Data Availability

The datasets used and/or analysed during this study are available from the corresponding author on reasonable request.

## Abbreviations

ADR: Adverse drug reaction
DDI: Drug-Drug Interaction
MCQ: Multi-Choice Question
PD: Pharmacodynamics
PE: Peer Evaluation
PK: Pharmacokinetics
