# Supplementary material for: Contributions of a blended learning based on peer evaluation for teaching drug-drug interactions to undergraduate pharmacy students
Source: BMC Med Educ. 2019 Nov 19;19:426. doi: 10.1186/s12909-019-1867-5 (PMC6862800; doi:10.1186/s12909-019-1867-5)
Supplement: Supplementary file 1 — Additional file 1. Examples of clinical cases. Two typical clinical cases related to pharmacokinetics and pharmacodynamics topics addressed by students during this training are presented. [file 12909_2019_1867_MOESM1_ESM.docx]

| Topics | Pharmacokinetics | | Pharmacodynamics |
| --- | --- | --- | --- |
| Clinical cases | **Example 1:** Management of DDI between atorvastatin and fluconazole, risk of rhabdomyolysis    Mrs Y, comes to the pharmacy to have her 72-year-old husband’s prescription renewed, as he suffers from hypertension and has hypercholesterolemia.  His treatment is the following:   - Bumetanide (BURINEX^®^) 1 mg, 1 tablet in the morning - Amlodipine (AMLOR^®^) 5 mg, 1capsule in the evening - Fluindione (PREVISCAN^®^) 20 mg, ¾ tablet in the evening - Atorvastatin (TAHOR^®^) 10 mg, 1 tablet in the evening - Potassium chloride (KALEORID^®^ LP) 1000 mg, 1 tablet in the morning   Before leaving, Mrs Y asks you for advice: her husband seems to have a fungus in his mouth because of his dental prosthesis and since she has TRIFLUCAN^®^ (fluconazole) drinkable suspension at home, she wants to know if she can give it to him to treat this fungus. She has already experienced the same type of problem herself and TRIFLUCAN^®^ was very effective.  **Example 2:** Management of DDI between lithium and non-steroidal anti-inflammatory drugs, risk of lithium toxicity  A 31-year-old patient comes to the pharmacy for the first time with this prescription:   - XEROQUEL^®^ LP 300 (quetiapine) 1tablet at bed time - TERALITHE^®^ LP 400 (lithium carbonate) 2 tablets in the evening - EFFEXOR^®^ LP 75 (venlafaxine) 2 tablets in the morning - THERALENE^®^ (alimemazine) oral drops 20 drops at bedtime   After the patient has been given the drugs, she asks for a box of NUROFENFLASH^®^ 400 mg (ibuprofen) for her headaches. | **Example 1:** Management of DDI between amiodarone and mizolastine, risk of QT interval prolongation  Mr X, 69 years old, has been complaining for a few weeks about being out of breath after only a small effort. His physician examines him, detects tachycardia and arrhythmia and decides to send him to a cardiologist. The cardiologist confirms the diagnosis and sets up the following treatment:   - Amiodarone (CORDARONE^®^) 200 mg, 2 tablets twice a day (morning and evening) for the first 5 days, then 1 tablet twice a day (morning and evening) for the long-term treatment - Bisoprolol (CARDENSIEL^®^) 2.5 mg; 1 tablet twice a day (morning and evening)   Mr X comes to the pharmacy with this prescription and asks you if he can continue the MIZOLLEN^®^ (mizolastine) treatment that he is used to taking every spring because his hay fever is bothering him again.  **Example 2:** Management of DDI between methadone and nalmefene, risk of relapse of an addiction  Max, 34 years old, consulted an addictologist two months ago for a heroin addiction problem. The doctor introduced a methadone syrup treatment that Max comes to the pharmacy daily to take  He has not taken heroin again.  His prescription is as follows:  Methadone syrup 40 mg/day (daily dispensation)  Today at the pharmacy, while he was taking his methadone, he told you that he is currently drinking a little too much alcohol “quite a few beers actually”. He would like to take a few SELINCRO^®^ (nalmefene) tablets which his roommate, who is treated for alcohol dependence would give him. | |
