# Supplementary material for: Contributions of a blended learning based on peer evaluation for teaching drug-drug interactions to undergraduate pharmacy students
Source: BMC Med Educ. 2019 Nov 19;19:426. doi: 10.1186/s12909-019-1867-5 (PMC6862800; doi:10.1186/s12909-019-1867-5)
Supplement: Supplementary file 2 — Additional file 2. Scoring grid (implemented in Moodle® platform). From this grid, peer evaluation mark obtained from each student under a total of 32 points and then reported under 20. PE-mark = [Σ (item mark* weight) * 20]/32 [file 12909_2019_1867_MOESM2_ESM.docx]

| **Aspects** | **Items** | **Evaluation** | **Marks** | **Weight** |
| --- | --- | --- | --- | --- |
| **1** | You would say that the work submitted by your peer is in a pleasant style to read (concise, presence of keywords, correct spelling) and that it respects the instructions imposed by the teachers. | Not at all | 0 | 1 |
|  |  | Partially | 1 |  |
|  |  | Totally | 2 |  |
| **2** | Your peers’ work presents the various descriptive elements of the patients’ pathophysiological profile that were present in the clinical case or can be inferred from it. | Several elements could have been added | 0 | 1 |
|  |  | Some elements could have been added | 1 |  |
|  |  | All the elements are presented | 2 |  |
| **3** | Did you miss any information on the pathology or situation or possibly any updated items in the reading of this work? | Several elements missing | 0 | 1 |
|  |  | Some elements missing | 1 |  |
|  |  | All were mentioned | 2 |  |
| **4** | Did the presentation allow you to easily understand the reasons for the drug prescription? Feel free to comment on what you may have missed. | Not at all | 0 | 1 |
|  |  | Partially | 1 |  |
|  |  | Totally | 2 |  |
| **5** | Was the description of the drugs correct (class, mechanism of action, spelling of INNs or specialities)? | Several mistakes | 0 | 1 |
|  |  | Some mistakes | 1 |  |
|  |  | Correct | 2 |  |
| **6** | Were all the elements about the expected adverse reactions or contraindications in regard to the physiological or pathological profile of the patient mentioned? | Not at all | 0 | 1 |
|  |  | Partially | 1 |  |
|  |  | Totally | 2 |  |
| **7** | In your opinion, has the level of the severity of the interaction (contraindication, association to be taken into account or safety precautions) been adequately taken into account? | Not at all | 0 | 3 |
|  |  | Partially | 1 |  |
|  |  | Totally | 2 |  |
| **8** | You would say that the arguments put forward by your peer to justify the mechanism(s) of the identified drug interactions are convincing and are based on bibliographic references or reference documents. | Not at all | 0 | 3 |
|  |  | Partially | 1 |  |
|  |  | Totally | 2 |  |
| **9** | You would say that what to do at the pharmacy in this context suggested by your peer sound appropriated or justified in regard to the gravity of the case. | Not adequate | 0 | 2 |
|  |  | Adequate | 1 |  |
|  |  | Exceptional | 2 |  |
| 10 | Messages or advices suggested to the patient were adapted. | No message or advice or not well adapted | 0 | 2 |
|  |  | Adapted | 1 |  |
|  |  | Well adapted | 2 |  |
