# Supplementary material for: Contributions of a blended learning based on peer evaluation for teaching drug-drug interactions to undergraduate pharmacy students
Source: BMC Med Educ. 2019 Nov 19;19:426. doi: 10.1186/s12909-019-1867-5 (PMC6862800; doi:10.1186/s12909-019-1867-5)
Supplement: Supplementary file 3 — Additional file 3. Online survey. Items of the survey completed by students after the training to evaluate relevance and usefulness of the blended learning and peer review approach in teaching drug-drug interactions. [file 12909_2019_1867_MOESM3_ESM.docx]

An original pedagogical activity based on the principle of peer evaluation has been proposed to you. In order to evaluate its relevance and usefulness in your training, your opinion is important. Thank you for spending a few minutes to complete this survey!

**Concerning the principle of peer review**

1. Peer review is used in science to evaluate the research works (e.g., selection of publications, funding research projects for funding).

Were aware of this? (Only one possible answer)

- Yes
- No

2. Peer evaluation is also used in pedagogy, particularly in the context of MOOC (Massive Open Online Course).

Have you ever heard of the principle of peer review applied to pedagogy? (Only one possible answer)

- Yes
- No

3. If yes, had you ever participated in training using this evaluation method? (Only one possible answer)

- Yes
- No

**Concerning the modalities of carrying out your peer review work**

4. Was the explanation provided to you prior to the course by instructors sufficient to understand the process? (Only one possible answer)

- Yes
- No

5. If not, what information did you miss?
6. Did you encounter any IT difficulties during the process? (Only one possible answer)

- Yes
- No

7. If yes, please specify the step concerned.
8. Was the time allowed for the completion of your assignment appropriate? (Only one possible answer)

- Yes
- No

9. If not, how long do you think it would have taken?
10. The number of assignments to be evaluated seemed to you to be: (Only one possible answer)

- Weak
- Adapted
- Too important

11. Was the time allowed to correct the duties of your peers appropriate? (Only one possible answer)

- Yes
- No

12. If not, how long do you think it would have taken?
13. At most, how many assignments could you have evaluated in the time allowed?
14. Was the scoring grid proposed by teachers consisting in awarding points for each aspect of the assignment appropriate? (Only one possible answer)

- Yes
- No

15. If not, what would you suggest to improve the scoring grid?
16. When performing the correction, did you make one or more of the following reflections? (Several possible answers)

- I am not legitimate to correct my peers
- My level of training is not sufficient to evaluate my peers
- It is up to the teacher to correct the work, not me!
- It is rewarding to be called upon as a reviewer
- Others reflections: (mentioned here)

17. The restitution stage (group work and oral presentation) seem to be: (Only one possible answer)

- Useless
- Not very useful
- Useful
- Indispensable

**Concerning your opinions about the course activities**

18. Did the activity of correcting your peers seem constraining to you? (Only one possible answer)

- Not at all constraining
- Constraining
- Very constraining

19. Do you feel that you have provided useful elements to your peers by correcting their work? (Only one possible answer)

- Not at all
- Perhaps
- Certainly

20. Do you feel that you have personally benefited from the corrective work done by your peers? (Only one possible answer)

- Not at all
- Perhaps
- Certainly

21. Do you feel that you have personally benefited from your work as a reviewer? (Only one possible answer)

- Not at all
- Perhaps
- Certainly

22. If this course activities wad beneficial to you, in what capacity? (Several possible answers)

- Helpful in memorizing knowledge
- Helpful in better understanding of knowledge
- Helpful in acquiring additional knowledge
- Helpful in questioning your own work
- Helpful in acquiring critical thinking skills
- No benefits
- Other benefits (mentioned here)

23. Which of the 3 phases has brought you the most benefit? (Several possible answers)

- Phase 1: the production of my assignment
- Phase 2: the evaluation of my peers’ work
- Phase 3: the group synthesis work
- Other phases (mentioned here)

24. On a scale ranging from 0 to 10, what is your overall feeling about this activity (Only one possible answer)

- 1 (poor)
- 2
- 3
- 4
- 5
- 6
- 7
- 8
- 9
- 10 (excellent)

25. Would you like this kind of activity to be proposed again during your pharmaceutical studies? (Only one possible answer)

- 1 (absolutely not)
- 2
- 3
- 4
- 5 (certainly)

If you wish, you can hereby give your global advices on this activity
